# The effect of medications associated with drug-induced pancreatitis on pancreatic cancer risk: A nested case-control study of routine Scottish data

**DOI:** 10.1016/j.canep.2020.101880

**Published:** 2021-04

**Authors:** R.D. McDowell, C.M. Hughes, P. Murchie, C.R. Cardwell

**Affiliations:** a Centre for Public Health, Queen’s University, Grosvenor Rd., Belfast, Co. Antrim, BT12 6 BA, UK; b School of Pharmacy, Queen’s University, Lisburn Rd., Belfast, Co. Antrim, BT9 7BL, UK; c Division of Applied Health Sciences Section, Academic Primary Care, Foresterhill, Aberdeen, AB24 2ZD, UK

**Keywords:** ACEI, Ace inhibitor, CCI, Charlson Comorbidity Index, CI, confidence interval, DIP, drug induced pancreatitis, FDA, Food and Drug Administration, GP, general practitioner, H2RA, histamine type-2 receptor agonist, MICE, multiple imputation with chained equations, NDMA, *N*-nitrosodimethylamine, PCCIUR, Primary Care Clinical Information Unit Research, OR, odds ratio, OTC, over-the-counter, Thin, The Health Improvement Network, UK, United Kingdom, Pancreatitis, Pancreatic neoplasms, Pharmacoepidemiology

## Abstract

**Background:**

Inflammation plays a role in pancreatic cancer. Many medications cause pancreatic inflammation, with some leading to a diagnosis of drug-induced pancreatitis (DIP), but few studies have examined these medications and pancreatic cancer risk. We therefore investigated the associations between pancreatic cancer risk and commonly-prescribed medicines for which there is strongest evidence of DIP.

**Methods:**

A nested case-control study was undertaken using the Primary Care Clinical Informatics Unit Research database containing general practice (GP) records from Scotland. Pancreatic cancer cases, diagnosed between 1999 and 2011, were identified and matched with up to five controls (based on age, gender, GP practice and date of registration). Medicines in the highest category of evidence for DIP, based on a recent systematic review, and used by more than 2 % of controls were identified. Odds ratios (OR) and 95 % confidence intervals (CI) for associations with pancreatic cancer were calculated using conditional logistic regression after adjusting for comorbidities.

**Results:**

There were 1,069 cases and 4,729 controls. Thirteen medicines in the highest category of evidence for DIP were investigated. There was little evidence of an association between any of these medications and pancreatic cancer risk apart from metronidazole (adjusted OR 1.69, 95 % CI 1.18, 2.41) and ranitidine (adjusted OR 1.37, 95 %CI 1.10, 1.70). However, no definitive exposure-response relationships between these medicines and cancer risk were observed.

**Conclusions:**

There is little evidence that commonly-prescribed medicines associated with inflammation of the pancreas are also associated with pancreatic cancer. These findings should provide reassurance to patients and prescribing clinicians.

## Highlights

• Inflammation plays a role in pancreatic cancer.
• Many medicines are known to cause inflammation of the pancreas.
• We studied medicines with the strongest evidence for drug-induced pancreatitis.
• Little evidence of associations between these medicines and pancreatic cancer.
• Medicines associated with pancreatitis not associated with pancreatic cancer.

## 1 Introduction

Pancreatic cancer is an aggressive form of cancer. Despite accounting for less than 3% (460,000) of new cases globally in 2018, pancreatic cancer was the seventh-leading cause of deaths from cancer (432,000) [1]. Incidence and mortality is particularly high in Europe, with over 95,000 deaths annually and a median survival of 4.6 months [2]. Pancreatic cancer is often diagnosed late stage and over the last forty years patient outcomes have not markedly improved [3] highlighting the importance of primary prevention.

It is widely accepted that inflammation is involved in the development of many cancers [4,5] due to the potential for cell mutation and proliferation as the body responds to tissue damage [6]. In particular, there is evidence of the role of inflammation in pancreatic cancer [7]. Chronic pancreatitis is a well-recognised risk factor for pancreatic cancer [8] due to interactions of pancreatic stellate cells with pancreatic cancer cells, acinar cells and inflammatory cells [9], and several studies have reported that acute pancreatitis is also associated with an increased risk of pancreatic cancer [[10], [11], [12]].

While gallstones [13] and alcohol consumption [14] are considered the most common precipitating factors for inflammation of the pancreas, there is increased awareness that medications can cause also inflammation which, although rare, can lead to the development of acute drug-induced pancreatitis (DIP) [15]. Due to an overreliance on clinician case reports and a lack of detailed information derived from large-scale population-based studies, DIP is considered a medical condition which can be both overdiagnosed and underdiagnosed [16]. Attempts have been made to systematically classify medications into those which have the strongest evidence of DIP and are most likely to cause inflammation of the pancreas. The number of medications identified as possible causes of DIP has increased from 60 in 2007 [15] to 214 in 2020 [17], and includes a wide variety of drugs such as diuretics [18], estrogens [19] and Angiotensin Converting Enzyme (ACE) inhibitors [20]. However, studies have not systematically investigated the association between these medications and pancreatic cancer risk. We therefore investigated the associations between pancreatic cancer and commonly-prescribed medicines associated with drug-induced pancreatitis using a nested case-control study.

## 2 Materials and methods

### 2.1 Data source

Data for this study was obtained from Primary Care Clinical Information Unit Research (PCCIUR) [21], a high quality population-based database of over two million patients registered at 393 general practices from across Scotland between 1993 and 2011. PCCIUR data contains up to 20 years of demographic, clinical and diagnostic information and has been widely used in epidemiological research [[22], [23], [24], [25]].

### 2.2 Study design

A historical nested case-control study was conducted using PCCIUR data, with data collected prospectively. Cases were patients with a new diagnosis of primary pancreatic cancer (Read code B17) between 1999 and 2011. Cases were excluded if they had a previous cancer, excluding non-melanoma skin cancer, or they were diagnosed with other primary cancers on the date of diagnosis due to uncertainty about the primary cancer and the potential for coding errors. All available controls (alive, registered with their GP and free from cancer (with the exception of non-melanoma skin cancer)) were identified for each case matching on practice, year of birth (plus or minus five years), gender and year of registration (in categories). Up to five controls for each case were randomly selected from those available, without replacement. The index date within each matched set was defined as the diagnosis date of pancreatic cancer in the case. Both cases and controls were required to have at least three years of follow-up data and remain registered with the same general practice over the follow-up period. Cases could be sampled as controls prior to diagnosis.

Within each matched set, the exposure period, i.e. the period of time over which medicine use was determined, started on either 1 January 1993 (as prescriptions before this time were less likely to be electronically recorded), or the most recent GP registration date if this occurred after January 1993. This ensured that all members within each matched set had the same exposure period. The exposure period ended one year before the index date, to reduce the risk of reverse causality and exclude medications that are unlikely to have had sufficient time to cause the cancer [26].

### 2.3 Classification and definition of medicine

The most recent and comprehensive systematic review of medicines associated with DIP [17] was used to identify medicines for study in relation to pancreatic cancer risk. This review classified evidence for the association between 240 medicines and DIP into one of six classes; from Class Ia (strongest evidence of an association), Class Ib, Class Ic, Class II, Class III, to Class IV (weakest evidence of an association). Medicines were assigned to Class Ia if there was at least one case report in humans with positive rechallenge (i.e. pancreatitis returned after stopping and restarting the drug) and all other causes such as alcohol, hypertriglyceridemia/hyperlipidemia, gallstones, and other medicines were ruled out. Systemic formulations of Class Ia medicines (single-agent drugs and the appropriate constituent parts of combination drugs) were extracted from the electronic GP prescribing records within PCCIUR. Analyses were restricted to Class Ia medicines that were issued to at least 2 percent of the controls.

### 2.4 Covariates

The following comorbidities, based upon published Read codes for the Charlson Comorbidity Index (CCI) [27], were identified prior to or during the exposure period: diabetes, myocardial infarction, coronary heart disease, heart failure, peripheral vascular disease, dementia, cerebrovascular disease, chronic obstructive pulmonary disease, osteoporosis, rheumatological disease, renal disease, liver disease, irritable bowel disease, human immunodeficiency viruses (HIV) and hemiplegia/paraplegia. Additional comorbidities, relevant to pancreatic cancer (peptic ulcer, *Helicobacter pylori,* Hepatitis B & C, gallstones, metabolic syndrome), were also identified. Smoking status (non-smoker, current smoker, former smoker) and alcohol consumption (non-drinker, light or moderate drinker, heavy drinker) were determined from the most recent smoking or alcohol record prior to or during the exposure period.

### 2.5 Statistical analysis

Descriptive statistics were used to summarise the cases and controls. Conditional logistic regression was used to calculate odds ratios (OR) and 95 % confidence intervals (CI) for the associations between each medicine and pancreatic cancer, with and without adjustment for comorbidities. The matched design accounted for age (+/- five years), GP practice, gender and year of registration. All analyses adjusted for age in years, allowing for the fact that patients were matched in age bands rather by calendar year. Exposure-response analyses were conducted calculating ORs for low and high use compared with none, with low/high use based upon numbers of prescriptions equal to or below/above the median (among the control patients who were users), respectively.

### 2.6 Sensitivity analyses

A number of sensitivity analyses were undertaken as follows: 1) the period of time before the index date during which prescriptions were not counted was increased from one year to two years to reduce the potential for reverse causation; 2) an exposure of > = 6 items of medicines (v less than 6 items) was used as a proxy for higher volume/repeat users; 3) adjustments were made for comorbidities, smoking and alcohol status for the 3,935 patients (67.9 %) with available data; 4) analyses adjusting for comorbidities, smoking and alcohol status were repeated using multiple imputation with chained equations (MICE) techniques to impute smoking and alcohol status. This is a simulation-based method appropriate for handling missing data when it is assumed that such values are missing at random or missing completely at random. Ordered logit models were used with age, gender, deprivation and comorbidities for the imputations, stratified by case-control status, and using 25 imputations [28].

All statistical analyses were undertaken using Stata 15 [29]. Results were held to be significant if they referred to statistical significance on a two-sided design-based test evaluated at the 0.05 % level.

## 3 Results

### 3.1 Descriptive statistics

1,069 cases and 4,729 controls were identified in the data. 914 cases (85.6 %) had at least 4 matched controls. The median exposure period was 8.9 years (Interquartile range (IQR) (6.0, 11.7)). The median age of all patients at diagnosis was 69 years (IQR 59–77) and 52.0 % (3,014) were female. The most commonly diagnosed comorbidities were coronary heart disease (952 (16.42 %)), diabetes (574 (9.9 %)) and chronic obstructive pulmonary disease (COPD) (449 (7.7 %). 389 (6.7 %) patients were diagnosed with gallstones. 35 out of a possible 45 Class Ia medicines were identified within PCCIUR, of which 13 were prescribed to at least 2% of the controls during the exposure period. Characteristics of the cases and controls are summarised in Table 1.

**Table 1:** Characteristics of cases and controls.

Variable	Category	Cases n (%)	Controls n (%)
		n = 1,069	n = 4,729
Length of exposure period (years): median (IQR)		8.9 (6.0,11.7)	8.9 (6.0,11.7)
Year of diagnosis/index date: median (IQR)		2006 (2003,2007)	2006 (2003, 2007)
Age at diagnosis/index date (years)	0−39	11 (1.1 %)	71 (1.5 %)
	40−59	198 (18.5 %)	1,179 (24.9 %)
	60−79	636 (59.5 %)	2,663 (56.3 %)
	80 +	224 (21.0 %)	816 (17.3 %)
Deprivation quintile	1 (least deprived)	67 (6.3 %)	287 (6.1 %)
	2	210 (19.6 %)	940 (19.9 %)
	3	84 (7.9 %)	370 (7.8 %)
	4	271 (25.4 %)	1,189 (25.1 %)
	5 (most deprived)	275 (25.7 %)	1,227 (25.95 %)
Gender	male	516 (48.3 %)	2,268 (48.0 %)
	female	553 (51.7 %)	2,461 (52.0 %)
Smoking status^a^	never smoked	338 (31.6 %)	1,800 (38.1 %)
	ex-smoker	278 (26.0 %)	1,115 (23.6 %)
	current smoker	287 (26.9 %)	867 (18.3 %)
	missing	166 (15.5 %)	947 (20.0 %)
Alcohol consumption^a^	non-drinker	179 (16.7 %)	747 (15.8 %)
	light/moderate	555 (51.9 %)	2,363 (50.0 %)
	heavy drinker	35 (3.3 %)	161 (3.4 %)
	missing	300 (28.1 %)	1,458 (30.8 %)

**Comorbidities diagnosed prior to or during the exposure period**			
Diabetes		178 (16.7 %)	396 (8.4 %)
Myocardial infarction		89 (8.3 %)	299 (6.3 %)
Coronary heart disease		212 (19.8 %)	740 (15.7 %)
Heart failure		49 (4.6 %)	143 (3.0 %)
Peripheral vascular disease		53 (5.0 %)	205 (4.3 %)
Dementia		9 (0.8 %)	70 (1.5 %)
Cerebrovascular disease		83 (7.8 %)	323 (6.8 %)
Chronic obstructive pulmonary disease		115 (10.8 %)	334 (7.1 %)
Osteoporosis		44 (4.1 %)	164 (6.5 %)
Rheumatological disease		44 (4.1 %)	121 (2.6 %)
Renal disease		48 (4.5 %)	221 (4.7 %)
Liver disease		17 (1.6 %)	26 (0.6 %)
Irritable bowel disease		58 (5.4 %)	287 (6.1 %)
Human immunodeficiency viruses		<5 (<0.5 %)	<5 (<0.1 %)
Hemiplegia/paraplegia		<5 (<0.5 %)	23 (0.5 %)
Peptic ulcer		95 (8.9 %)	354 (7.5 %)
*Helicobacter pylori*		<5 (<0.5 %)	<5 (<0.1 %)
Hepatitis B/C		<5 (<0.5 %)	<5 (<0.1 %)
Gallstones		89 (8.3 %)	300 (6.3 %)
Metabolic syndrome		<5 (<0.5 %)	<5 (<0.1 %)

Abbreviations: IQR: inter-quartile range; DIP: drug induced pancreatitis.

a Most recent record in patient’s clinical history prior to one-year lag.

### 3.2 Associations between medicines and pancreatic cancer

**Fig. 1:**
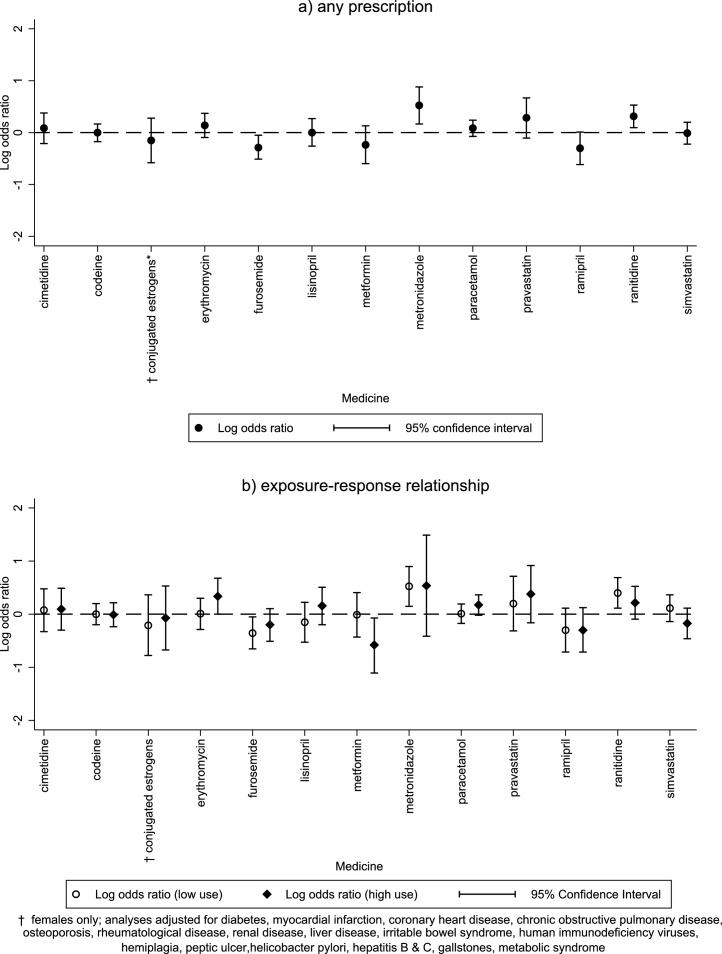
Associations of commonly-prescribed Class Ia medicines with pancreatic cancer .

Results from the main analyses are illustrated in Fig. 1 and reported in Table 2. Overall, the majority of Class Ia medicines were not associated with an increased risk of pancreatic cancer after adjustment for comorbid conditions. Only two medicines were significantly associated with an increased risk of pancreatic cancer (metronidazole: adjusted odds ratio (OR_adj_) 1.69, 95 % CI (1.18, 2.41), p = 0.004; ranitidine: OR_adj_ 1.37, 95 %CI (1.10,1.70), p = 0.005). However, neither exhibited strong evidence of an exposure-response relationship with cancer risk. For ranitidine, the adjusted odds ratio comparing six or more prescriptions with none was 1.24 (95 % CI 0.91, 1.69; p = 0.179), whereas the adjusted odds ratio comparing five or fewer prescriptions with none was 1.49 (95 %CI 1.12.1.99; p = 0.007). For metronidazole, the adjusted odds ratio comparing two or more prescriptions with none was 1.71 (95 %CI 0.66, 4.43; p = 0.272), whereas the adjusted odds ratio comparing one prescription with none was 1.69 (95 %CI 1.16, 2.45; p = 0.006). Although there was little association with any use or erythromycin and pancreatic cancer (OR_adj_ 1.15, 95 %CI (0.91, 1.45), p = 0.238), there was evidence that patients issued two or more erythromycin prescriptions were associated with higher pancreatic cancer risk (OR_adj_ 1.40, 95 %CI (1.00, 1.97), p = 0.049).

**Table 2:** Associations of commonly-prescribed Class Ia medicines with pancreatic cancer.

Medicine	Category	No (%) cases	No (%) controls	Unadjusted OR (95 %CI)	Comorbidity^a^ adjusted OR (95%CI)
cimetidine	Never	995 (93.1 %)	4,448 (94.1 %)	1.00	1.00
	Any	74 (6.9 %)	281 (5.9 %)	1.13 (0.85,1.50)	1.09 (0.81,1.46)
	Lower usage (1−3)	35 (3.3 %)	144 (3.0 %)	1.06 (0.72,1.57)	1.08 (0.72,1.61)
	Higher usage (> = 4)	39 (3.7 %)	137 (2.9 %)	1.20 (0.82,1.76)	1.10 (0.74,1.63)

codeine	Never	738 (69.0 %)	3,343 (70.7 %)	1.00	1.00
	Any	331 (31.0 %)	1,386 (29.3 %)	1.06 (0.90,1.24)	1.00 (0.84,1.18)
	Lower usage (1−3)	176 (16.5 %)	750 (15.9 %)	1.05 (0.87,1.28)	1.00 (0.82,1.22)
	Higher usage (> = 4)	155 (14.5 %)	636 (13.4 %)	1.07 (0.86,1.32)	0.99 (0.79,1.24)

conjugated estrogens^b^	Never	522 (94.4 %)	2,269 (92.2 %)	1.00	1.00
	Any	31 (5.6 %)	192 (7.8 %)	0.83 (0.54,1.26)	0.86 (0.56,1.32)
	Lower usage (1−6)	16 (2.9 %)	102 (4.1 %)	0.80 (0.45,1.40)	0.81 (0.46,1.44)
	Higher usage (> = 7)	15 (2.7 %)	90 (3.7 %)	0.86 (0.48,1.56)	0.93 (0.51,1.70)

erythromycin	Never	943 (88.2 %)	4,247 (89.8 %)	1.00	1.00
	Any	126 (11.8 %)	482 (10.2 %)	1.27 (1.02,1.59)	1.15 (0.91,1.45)
	Lower usage (1−1)	67 (6.3 %)	318 (6.7 %)	1.06 (0.80,1.41)	1.01 (0.75,1.35)
	Higher usage (> = 2)	59 (5.5 %)	164 (3.5 %)	1.67 (1.21,2.32)	1.40 (1.00,1.97)

furosemide	Never	924 (86.4 %)	4,137 (87.5 %)	1.00	1.00
	Any	145 (13.6 %)	592 (12.5 %)	0.96 (0.78,1.19)	0.75 (0.60,0.95)
	Lower usage (1−13)	65 (6.1 %)	303 (6.4 %)	0.88 (0.66,1.17)	0.70 (0.52,0.95)
	Higher usage (> = 14)	80 (7.5 %)	289 (6.1 %)	1.05 (0.80,1.38)	0.82 (0.60,1.11)

lisinopril	Never	978 (91.5 %)	4,390 (92.8 %)	1.00	1.00
	Any	91 (8.5 %)	339 (7.2 %)	1.12 (0.87,1.46)	1.00 (0.77,1.31)
	Lower usage (1−12)	40 (3.7 %)	175 (3.7 %)	0.93 (0.65,1.34)	0.86 (0.59,1.25)
	Higher usage (> = 13)	51 (4.8 %)	164 (3.5 %)	1.35 (0.96,1.90)	1.17 (0.82,1.66)

metformin	Never	984 (92.0 %)	4,521 (95.6 %)	1.00	1.00
	Any	85 (8.0 %)	208 (4.4 %)	1.93 (1.47,2.53)	0.79 (0.55,1.14)
	Lower usage (1−23)	58 (5.4 %)	108 (2.3 %)	2.47 (1.76,3.45)	0.99 (0.65,1.50)
	Higher usage (> = 24)	27 (2.5 %)	100 (2.1 %)	1.32 (0.85,2.05)	0.56 (0.33,0.93)

metronidazole	Never	1,017 (95.1 %)	4,586 (97.0 %)	1.00	1.00
	Any	52 (4.9 %)	143 (3.0 %)	1.73 (1.23,2.43)	1.69 (1.18,2.41)
	Lower usage (1−1)	46 (4.3 %)	121 (2.6 %)	1.75 (1.22,2.50)	1.69 (1.16,2.45)
	Higher usage (> = 2)	6 (0.6 %)	22 (0.5 %)	1.59 (0.63,4.03)	1.71 (0.66,4.43)

paracetamol	Never	523 (48.9 %)	2,566 (54.3 %)	1.00	1.00
	Any	546 (51.1 %)	2,163 (45.7 %)	1.19 (1.02,1.38)	1.09 (0.93,1.27)
	Lower usage (1−6)	253 (23.7 %)	1,118 (23.6 %)	1.08 (0.91,1.29)	1.01 (0.84,1.21)
	Higher usage (> = 7)	293 (27.4 %)	1,045 (22.1 %)	1.32 (1.10,1.59)	1.19 (0.98,1.44)

pravastatin	Never	1,024 (95.8 %)	4,594 (97.2 %)	1.00	1.00
	Any	45 (4.2 %)	135 (2.9 %)	1.54 (1.07,2.22)	1.33 (0.90,1.95)
	Lower usage (1−14)	22 (2.1 %)	69 (1.5 %)	1.42 (0.87,2.33)	1.22 (0.73,2.04)
	Higher usage (> = 15)	23 (2.1 %)	66 (1.4 %)	1.68 (1.01,2.81)	1.46 (0.85,2.50)

ramipril	Never	1,003 (93.8 %)	4,453 (94.2 %)	1.00	1.00
	Any	66 (6.2 %)	276 (5.8 %)	0.98 (0.73,1.31)	0.74 (0.54,1.01)
	Lower usage (1−12)	32 (3.0 %)	141 (3.0 %)	0.94 (0.63,1.40)	0.74 (0.49,1.12)
	Higher usage (> = 13)	34 (3.2 %)	135 (2.9 %)	1.03 (0.69,1.52)	0.74 (0.49,1.13)

ranitidine	Never	926 (86.6 %)	4,287 (90.7 %)	1.00	1.00
	Any	143 (13.4 %)	442 (9.3 %)	1.45 (1.17,1.79)	1.37 (1.10,1.70)
	Lower usage (1−5)	79 (7.4 %)	230 (4.9 %)	1.61 (1.22,2.12)	1.49 (1.12,1.99)
	Higher usage (> = 6)	64 (6.0 %)	212 (4.5 %)	1.28 (0.95,1.73)	1.24 (0.91,1.69)

simvastatin	Never	862 (80.6 %)	3,975 (84.1 %)	1.00	1.00
	Any	207 (19.4 %)	754 (15.9 %)	1.23 (1.02,1.48)	0.99 (0.80,1.22)
	Lower usage (1−14)	117 (10.9 %)	381 (8.1 %)	1.38 (1.09,1.74)	1.12 (0.87,1.44)
	Higher usage (> = 15)	90 (8.4 %)	373 (7.9 %)	1.07 (0.83,1.39)	0.84 (0.63,1.12)

Abbreviations: OR: odds ratio; CI: confidence interval.

a Comorbidities include diabetes, myocardial infarction, coronary heart disease, heart failure, peripheral vascular disease, dementia, cerebrovascular disease, chronic obstructive pulmonary disease, osteoporosis, rheumatological disease, renal disease, liver disease, irritable bowel disease, human immunodeficiency viruses, hemiplegia/paraplegia, peptic ulcer, *helicobacter pylori*, Hepatitis B/C, gallstones, metabolic syndrome.

b Females only.

The results from the sensitivity analyses are listed in Table 3. Increasing the lag-time from one year to two years or adjusting for smoking and alcohol use had no substantive impact on the reported associations between prescribed medicines and risk of pancreatic cancer. It was not possible to estimate an odds ratio for the association between six or more items of metronidazole and pancreatic cancer as all cases were issued with less than six metronidazole prescriptions during the exposure period.

**Table 3:** Sensitivity analyses for associations of commonly-prescribed Class Ia medicines and pancreatic cancer.

Medicine	Analysis	Exposure	No (%) cases	No (%) controls	Unadjusted OR (95 %CI)	Comorbidity a adjusted OR (95%CI)
cimetidine	2 year lag	Any	70(6.6 %)	267(5.7 %)	1.12(0.84,1.49)	1.07(0.79,1.45)
	Lifestyle complete b	Any	51(6.7 %)	215(6.8 %)	0.96(0.67,1.37)	0.91(0.63,1.32)
	Lifestyle MI b	Any	74(6.9 %)	281(5.9 %)	1.13(0.85,1.50)	1.07(0.80,1.43)
	1 year lag	> = 6 items	31(2.9 %)	119(2.5 %)	1.07(0.71,1.63)	0.98(0.64,1.51)

codeine	2 year lag	Any	278(26.0 %)	1,189(25.1 %)	1.02(0.86,1.21)	0.96(0.80,1.14)
	Lifestyle complete b	Any	269(35.4 %)	1,097(34.5 %)	1.06(0.87,1.29)	0.98(0.80,1.21)
	Lifestyle MI b	Any	331(31.0 %)	1,386(29.3 %)	1.06(0.90,1.24)	0.99(0.84,1.17)
	1 year lag	> = 6 items	128(12.0 %)	517(10.9 %)	1.07(0.85,1.33)	1.00(0.79,1.26)

conjugated estrogens c	2 year lag	Any	30(5.4 %)	187(7.6 %)	0.83(0.54,1.27)	0.86(0.56,1.32)
	Lifestyle complete b	Any	26(6.6 %)	161(9.5 %)	0.79(0.49,1.28)	0.80(0.49,1.31)
	Lifestyle MI b	Any	31(5.6 %)	192(7.8 %)	0.83(0.54,1.26)	0.83(0.54,1.27)
	1 year lag	> = 6 items	15(2.7 %)	100(4.1 %)	0.76(0.42,1.38)	0.78(0.43,1.43)

erythromycin	2 year lag	Any	115(10.8 %)	432(9.1 %)	1.28(1.02,1.61)	1.16(0.91,1.47)
	Lifestyle complete b	Any	105(13.8 %)	381(12.0 %)	1.28(0.99,1.66)	1.13(0.86,1.48)
	Lifestyle MI b	Any	126(11.8 %)	482(10.2 %)	1.27(1.02,1.59)	1.17(0.93,1.48)
	1 year lag	> = 6 items	7(0.7 %)	18(0.4 %)	1.71(0.70,4.21)	1.63(0.65,4.11)

furosemide	2 year lag	Any	125(11.7 %)	529(11.2 %)	0.91(0.73,1.14)	0.71(0.56,0.91)
	Lifestyle complete b	Any	117(15.4 %)	432(13.6 %)	0.97(0.75,1.26)	0.74(0.55,0.99)
	Lifestyle MI b	Any	145(13.6 %)	592(12.5 %)	0.96(0.78,1.19)	0.76(0.60,0.96)
	1 year lag	> = 6 items	96(9.0 %)	406(8.6 %)	0.89(0.69,1.14)	0.70(0.53,0.92)

lisinopril	2 year lag	Any	71(6.6 %)	280(5.9 %)	1.07(0.80,1.43)	0.93(0.69,1.26)
	Lifestyle complete b	Any	82(10.8 %)	274(8.6 %)	1.20(0.89,1.61)	1.08(0.79,1.48)
	Lifestyle MI b	Any	91(8.5 %)	339(7.2 %)	1.12(0.87,1.46)	1.03(0.79,1.36)
	1 year lag	> = 6 items	64(6.0 %)	233(4.9 %)	1.14(0.85,1.55)	1.02(0.75,1.39)

metformin	2 year lag	Any	70(6.6 %)	180(3.8 %)	1.86(1.38,2.49)	0.78(0.53,1.13)
	Lifestyle complete b	Any	75(9.9 %)	184(5.8 %)	1.92(1.40,2.62)	0.76(0.50,1.17)
	Lifestyle MI b	Any	85(8.0 %)	208(4.4 %)	1.93(1.47,2.53)	0.83(0.58,1.21)
	1 year lag	> = 6 items	68(6.4 %)	167(3.5 %)	1.93(1.44,2.61)	0.84(0.58,1.23)

metronidazole	2 year lag	Any	43(4.0 %)	121(2.6 %)	1.69(1.17,2.46)	1.69(1.15,2.49)
	Lifestyle complete b	Any	43(5.7 %)	114(3.6 %)	1.93(1.29,2.87)	1.91(1.25,2.91)
	Lifestyle MI b	Any	52(4.9 %)	143(3.0 %)	1.73(1.23,2.43)	1.68(1.18,2.41)
	1 year lag	> = 6 items	0(0.0 %)	1(0.0 %)	–	–

paracetamol	2 year lag	Any	496(46.4 %)	1,964(41.5 %)	1.16(1.00,1.35)	1.07(0.92,1.25)
	Lifestyle complete b	Any	424(55.8 %)	1,624(51.1 %)	1.20(0.99,1.45)	1.08(0.88,1.32)
	Lifestyle MI b	Any	546(51.1 %)	2,163(45.7 %)	1.19(1.02,1.38)	1.07(0.91,1.25)
	1 year lag	> = 6 items	308(28.8 %)	1,102(23.3 %)	1.28(1.09,1.51)	1.19(1.00,1.41)

pravastatin	2 year lag	Any	43(4.0 %)	123(2.6 %)	1.63(1.12,2.38)	1.42(0.96,2.11)
	Lifestyle complete b	Any	40(5.3 %)	119(3.8 %)	1.40(0.93,2.10)	1.28(0.82,1.98)
	Lifestyle MI b	Any	45(4.2 %)	135(2.9 %)	1.54(1.07,2.22)	1.33(0.90,1.96)
	1 year lag	> = 6 items	33(3.1 %)	102(2.2 %)	1.47(0.96,2.23)	1.28(0.82,1.99)

ramipril	2 year lag	Any	48(4.5 %)	223(4.7 %)	0.88(0.63,1.23)	0.63(0.44,0.90)
	Lifestyle complete b	Any	60(7.9 %)	237(7.5 %)	0.95(0.69,1.31)	0.72(0.50,1.02)
	Lifestyle MI b	Any	66(6.2 %)	276(5.8 %)	0.98(0.73,1.31)	0.74(0.54,1.01)
	1 year lag	> = 6 items	45(4.2 %)	193(4.1 %)	0.96(0.68,1.36)	0.70(0.49,1.01)

ranitidine	2 year lag	Any	129(12.1 %)	412(8.7 %)	1.39(1.12,1.74)	1.32(1.05,1.66)
	Lifestyle complete b	Any	115(15.1 %)	320(10.1 %)	1.48(1.14,1.93)	1.37(1.05,1.81)
	Lifestyle MI b	Any	143(13.4 %)	442(9.3 %)	1.45(1.17,1.79)	1.35(1.08,1.68)
	1 year lag	> = 6 items	64(6.0 %)	212(4.5 %)	1.24(0.92,1.68)	1.20(0.88,1.63)

simvastatin	2 year lag	Any	165(15.4 %)	625(13.2 %)	1.16(0.95,1.42)	0.91(0.73,1.15)
	Lifestyle complete b	Any	184(24.2 %)	650(20.5 %)	1.12(0.90,1.39)	0.93(0.73,1.19)
	Lifestyle MI b	Any	207(19.4 %)	754(15.9 %)	1.23(1.02,1.48)	0.99(0.80,1.22)
	1 year lag	> = 6 items	152(14.2 %)	567(12.0 %)	1.16(0.94,1.43)	0.93(0.74,1.18)

Abbreviations: OR: odds ratio; CI: confidence interval; MI: multiple imputation.

a Comorbidities include diabetes, myocardial infarction, coronary heart disease, heart failure, peripheral vascular disease, dementia, cerebrovascular disease, chronic obstructive pulmonary disease, osteoporosis, rheumatological disease, renal disease, liver disease, irritable bowel disease, human immunodeficiency viruses, hemiplegia/paraplegia, peptic ulcer, *helicobacter pylori*, Hepatitis B & C, gallstones, metabolic syndrome.

b Additionally adjusted for smoking and alcohol status.

c Females only.

## 4 Discussion

### 4.1 Principal findings

In this study we used a population-based clinical database to examine associations between commonly-prescribed medicines associated with inflammation of the pancreas and pancreatic cancer. Of the 13 medicines studied, two (metronidazole, ranitidine) were significantly associated with an increased risk of pancreatic cancer. However, there was little evidence of an exposure-response relationship with pancreatic cancer for these medicines. There was some evidence of an increased risk with pancreatic cancer for patients who received a greater number of prescriptions for erythromycin. The remaining medicines were not associated with an altered risk of pancreatic cancer after adjusting for comorbidities.

### 4.2 Context of other studies

To our knowledge this is the first study which has examined associations between a wide range of medications which have been shown to cause inflammation of the pancreas including acute pancreatitis and pancreatic cancer risk. Previous studies of medicine/pancreatic cancer associations have tended to study individual medicines [30] or medicines from the same family [31].

The medicines we studied were identified from a systematic review of the evidence of their impact on drug-induced pancreatitis and have previously been classed as having the strongest evidence of causing DIP. In our analyses use of ranitidine, a histamine type-2 receptor agonist (H2RA), was associated with a significantly increased risk of pancreatic cancer although no exposure-response relationship was observed. Concerns about the identification of low levels in ranitidine of *N*-nitrosodimethylamine (NDMA), a probable human carcinogen, led the Food and Drug Administration (FDA) in the United States in 2019 to withdraw all products which include ranitidine [32]. However a recent study of over 65 million American adults reported that users of ranitidine have a lower risk of pancreatic cancer than users of famotidine (OR_adj_ 0.63, 95 %CI (0.61, 0.65)), another H_2_RA commonly prescribed for the treatment of excess stomach acid [33]. In supplementary analyses of PCCIUR data neither famotidine (OR_ad_ 2.71, 95 %CI (0.41, 17.81), p = 0.300) nor nizatidine (OR_ad_ 1.01, 95 %CI (0.40,2.54), p = 0.979 were significantly associated with cancer risk. Findings from these two studies may vary for a variety of reasons such as sampling variation, differences in the study populations (e.g. age distributions), relatively lower levels of prescribing of famotidine in Scotland, and different adjustments for confounding variables. As such the association between ranitidine and pancreatic cancer is yet to be determined.

No significant association was observed in our study between cimetidine, another H_2_RA, and pancreatic cancer. Although a Danish study of patients prescribed cimetidine as a treatment for gastric ulcer observed an increased risk of pancreatic cancer in the first year of follow-up, they concluded this was unlikely to be due to any carcinogenic action of the drug [34]. More recently cimetidine has been shown to exhibit anti-tumour action via a number of mechanisms, such as reducing cancer cell proliferation, immunomodulation, cell adhesion and angiogenesis [35].

We observed that metronidazole, a nitroimidazole antibiotic, was associated with a higher risk of pancreatic cancer, as were patients with a greater number of erythromycin (a mitochondrially-targeted antibiotic) prescriptions. We cannot infer much about any potential causal relationship between either antibiotic and pancreatic cancer use to low numbers of patients using two or more prescriptions making an exposure-response relationship impossible to investigate. These results may reflect residual confounding; metronidazole is prescribed to reduce infection, such as reducing the risk of developing infected pancreatic necrosis in patients with necrotizing pancreatitis (i.e. where part of the pancreas dies) [36].

Metformin is the Class Ia medicine most commonly studied in relation to pancreatic cancer risk. However, results from pharmacoepidemiologic studies are varied. A nested case-control study of 529 pancreatic cancer cases and 5,000 controls from The Health Improvement Network (THIN) reported that metformin was associated with increased pancreatic cancer risk [37]. Bodmer et al. reported that use of metformin was associated with a decreased risk of pancreatic cancer in women only [30], whereas Walker et al. reported that there was no association between metformin and pancreatic cancer among patients with type 2 diabetes [38]. The potential of metformin, an antidiabetic drug, to have cancer risk-preventing properties has been known for many years [39]. Although metformin use was associated with a decreased risk of pancreatic cancer in our study, after adjusting for comorbidities, including diabetes, this association was not of statistical significance.

The remainder of our results are generally consistent with the literature; an increased risk of pancreatic cancer was not observed with use of hormone replacement therapy (HRT) medicines [40,41], paracetamol [31] or statins [42,43].

### 4.3 Strengths and limitations of study

There are a number of strengths to our study. The PCCIUR is a nationally-representative database, covering 15 % of Scottish patients. The linking of practice data to Scottish Cancer Registry data means there is a high coverage of cancer cases and a relatively long exposure period (approximately nine years). We used the most recent systematic review of medicines associated with DIP and analysed medicines which are commonly prescribed and for which there is the strongest clinical evidence of an association with drug induced pancreatitis, such as the elimination of other causes and presentation after re-challenge. Class Ia medicines not identified in our sample are either not currently licensed or are rarely prescribed within the United Kingdom (UK). We were able to adjust for a wide range of confounders.

However, there are inevitably limitations to this study. We did not have access to any secondary care prescribing details, such as hospital discharge summaries. However, if a medicine was initiated in secondary care for long-term use, we would expect it to be identified in subsequent GP prescribing records. Another limitation is our inability to account for over-the-counter (OTC) purchases of medicines. Ranitidine, for example, was licensed for OTC purchase in the UK January 1995 [44], but is only available OTC for short-term use (less than 2 weeks), at low doses (75 mg) and for limited indications (short-term symptomatic relief of heartburn, dyspepsia and hyperacidity) [45]. A previous methodological study has shown that healthcare datasets can produce valid estimates of medications despite OTC use [46].

We do not know the extent to which patients took their medicines, although results using an exposure of six or more items, as a proxy for longer-term use, were similar to those with an exposure of any medicine. We were unable to control for body mass index or other risk factors (such as genetic risk factors) as these were not recorded for the majority of PCCIUR patients, and some of the associations observed may be due to residual confounding. Finally, it is possible that the inclusion of all users of a given medicine during the exposure period, rather than new users, may result in the misclassification of drug use due to not counting medicines prescribed prior to the start of the exposure period [47]. This is a necessary component of the study design in order to make a fair comparison between cases and their matched controls. A new-user design, which only includes patients who begin a course of medicine, reduces this risk, but may be of limited value if long-term or historical medicine use is thought to alter cancer risk [48].

### 4.4 Implications for policy and research

Many of the medicines investigated are prescribed long-term for common medical conditions. Our study suggests that any inflammation of the pancreas which arises from taking these medicines does not appear to be associated with an increased risk of pancreatic cancer. These findings should provide some reassurance to patients who take these medicines for other conditions. However, given the increased risk of pancreatic cancer associated with ranitidine in our study, and current controversies concerning NDMA, we recommend that further studies of the association between ranitidine and pancreatic cancer take place over time-frames and/or in countries where ranitidine was only available on prescription.

## 5 Conclusions

In our study, medications recently classified as having the strongest evidence for causing drug-induced pancreatitis did not appear to be associated with pancreatic cancer. These findings should provide reassurance to patients regularly taking these medicines and doctors who are prescribing them.

## Ethical approval & consent to participate

The study was approved by the Research Applications and Data Management Team at the University of Aberdeen and Queen’s University Belfast, School of Medicine, Ethics Committee (reference number: 18.02v2). According to Caldicott principles patients registered at participating practices were made aware that their anonymous data could be used in research, and had the opportunity to withdraw should they wish.

## Consent for publication

Not applicable.

## Funding

This work was supported by 10.13039/501100000289Cancer Research UK (reference C37316/A25535).

## Availability of data and materials

The datasets analysed in this study are not publicly available and were used under license. Requests for PCCIUR data should be directed in the first instance to Katie Wilde (Research Manager), email: k.wilde@abdn.ac.uk.

## CRediT authorship contribution statement

**R.D. McDowell:** Conceptualization, Data curation, Formal analysis, Methodology, Project administration, Software, Visualization, Writing - original draft, Writing - review & editing. **C.M. Hughes:** Conceptualization, Funding acquisition, Resources, Supervision, Writing - review & editing. **P. Murchie:** Conceptualization, Funding acquisition, Resources, Supervision, Writing - review & editing. **C.R. Cardwell:** Conceptualization, Funding acquisition, Project administration, Resources, Supervision, Validation, Writing - original draft, Writing - review & editing.

## Declaration of Competing Interest

The authors report no declarations of interest.
